# Anti‐bacterial and wound healing‐promoting effects of zinc ferrite nanoparticles

**DOI:** 10.1186/s12951-021-00776-w

**Published:** 2021-02-05

**Authors:** Reihaneh Haghniaz, Atiya Rabbani, Fereshteh Vajhadin, Taous Khan, Rozina Kousar, Abdul Rehman Khan, Hossein Montazerian, Javed Iqbal, Alberto Libanori, Han-Jun Kim, Fazli Wahid

**Affiliations:** 1 Department of Bioengineering, University of California, Los Angeles, Los Angeles, CA 90095 USA; 2 California NanoSystems Institute (CNSI), University of California, Los Angeles, Los Angeles, CA 90095 USA; 3 Department of Biotechnology, COMSATS University Islamabad, Islamabad, 45550 Pakistan; 4 Department of Chemistry, Yazd University, 89195-741 Yazd, Iran; 5 Department of Pharmacy, COMSATS University Islamabad, Islamabad, 45550 Pakistan; 6 Department of Pharmacy, Women Institute of Learning, Abbottabad, 22060 Pakistan; 7 Department of Botany, Bacha Khan University, Charsadda, 24420 Pakistan; 8 Terasaki Institute for Biomedical Innovation, Los Angeles, CA 90064 USA; 9 Department of Biomedical Sciences, Pak-Austria Fachhochschule: Institute of Applied Sciences and Technology, Haripur, 22620 Pakistan

**Keywords:** Nanoparticles, Spinel ferrites, Zinc ferrites, Antimicrobial activity, Wound healing, Biocompatibility, Hemocompatibility, Antibiotics

## Abstract

**Background:**

Increasing antibiotic resistance continues to focus on research into the discovery of novel antimicrobial agents. Due to its antimicrobial and wound healing-promoting activity, metal nanoparticles have attracted attention for dermatological applications. This study is designed to investigate the scope and bactericidal potential of zinc ferrite nanoparticles (ZnFe_2_O_4_ NPs), and the mechanism of anti-bacterial action along with cytocompatibility, hemocompatibility, and wound healing properties.

**Results:**

ZnFe_2_O_4_ NPs were synthesized via a modified co-precipitation method. Structure, size, morphology, and elemental compositions of ZnFe_2_O_4_ NPs were analyzed using X-ray diffraction pattern, Fourier transform infrared spectroscopy, and field emission scanning electron microscopy coupled with energy-dispersive X-ray spectroscopy. In PrestoBlue and live/dead assays, ZnFe_2_O_4_ NPs exhibited dose-dependent cytotoxic effects on human dermal fibroblasts. In addition, the hemocompatibility assay revealed that the NPs do not significantly rupture red blood cells up to a dose of 1000 µg/mL. Bacterial live/dead imaging and zone of inhibition analysis demonstrated that ZnFe_2_O_4_ NPs showed dose-dependent bactericidal activities in various strains of Gram-negative and Gram-positive bacteria. Interestingly, NPs showed antimicrobial activity through multiple mechanisms, such as cell membrane damage, protein leakage, and reactive oxygen species generation, and were more effective against gram-positive bacteria. Furthermore, in vitro scratch assay revealed that ZnFe_2_O_4_ NPs improved cell migration and proliferation of cells, with noticeable shrinkage of the artificial wound model.

**Conclusions:**

This study indicated that ZnFe_2_O_4_ NPs have the potential to be used as a future antimicrobial and wound healing drug.
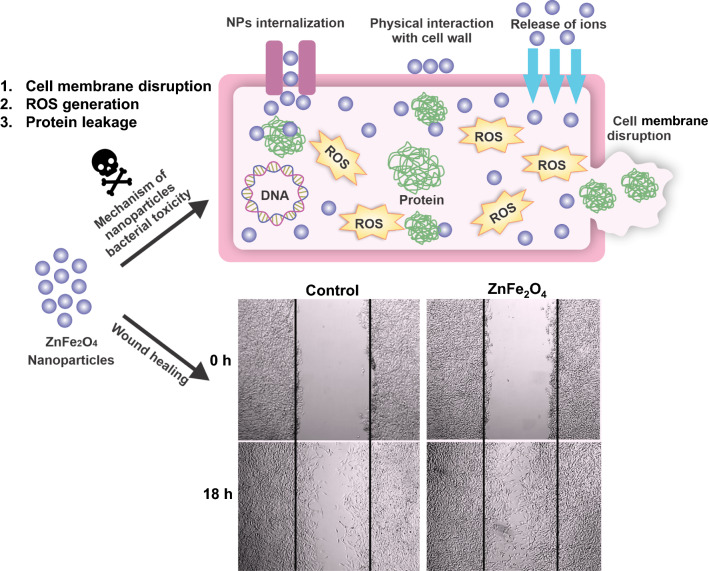

## Background

Since the accidental discovery of penicillin, antibiotics have been used to treat infectious diseases, which has significantly increased life expectancy and reduced mortality. However, due to excessive use and mismanagement of antibiotics, microorganisms develop resistance to a variety of traditional antibiotics over time, presenting major health problems for future infectious disease management [1, 2]. Recently, deaths associated with bacterial infection are increasing, due to the limited efficacy of existing antibiotics against certain microorganisms, such as methicillin-resistant *Staphylococcus aureus* (MRSA), *Streptococcus Pneumoniae*, and carbapenem-resistant *Enterobacteriaceae*. Therefore, new antibiotic treatment strategies are needed that can effectively treat bacterial infections that are resistant to existing antibiotics [3].

Burn wounds are the most lethal form of trauma that requires infection control to reduce morbidity and mortality [4]. Severe thermal damage results in damage to the skin’s superficial barrier as well as an immunosuppressive state, making burn patients vulnerable to infectious complications [5]. According to the previous clinical studies, 42–65 % of deaths amongst burn patients are due to infections [6]. The resistance of burn wound-related pathogens such as *Staphylococcus aureus* (*S. aureus*) and *Pseudomonas aeruginosa* (*P. aeruginosa*) to local and systemic anti-infective agents is making treatment more difficult [7]. Therefore, for the management of burn wound infection, new antimicrobial agents that are effective against antibiotic-resistant bacteria, non-toxic to normal cells, cost-effective, and do not cause bacterial resistance are needed.

The use of inorganic nanoparticles particularly metal nanoparticles (MNPs) and their oxides (e.g., ZnO [8], AgO [9], CuO [10], and CoO [11] NPs) has gained significant attention because of their promising results in targeted drug delivery [12], medical imaging [13], cancer treatment [14, 15], and inhibiting bacterial growth [16, 17] as well as promoting wound healing potential [18]. Furthermore, MNPs are desirable in fungicidal [19], and antimicrobial pharmaceuticals due to their durability, high stability, and low mammalian cell toxicity compared to organic NPs. Their nanoscale size and variable surface chemistry allow them to induce bacterial toxicity through several modes of actions such as, lipid peroxidation, oxidative stress, cell membrane lysis, enzyme inhibition, and proteolysis [17, 20, 21].

Among different MNPs, iron oxide-based NPs, especially spinel ferrites, have recently emerged as popular candidates for emerging biomedical applications due to their chemical stability, biocompatibility, the reasonable cost compared to the other MNPs such as silver NPs (i.e., gold standard anti-bacterial NPs), and their unique ferromagnetic properties [22, 23]. Spinel ferrites are homogenous materials consisting primarily of iron and have a general chemical formula of MFe_2_O_4_, in which M^2+^ and Fe^3+^ respectively reside in tetrahedral and octahedral metallic cation spots of the lattice [22]. The minuscule dimension of spinel ferrite NPs makes them an ideal candidate in a broad spectrum of biological applications, including their use as contrast agents in magnetic resonance imaging (MRI) [24], targeted drug delivery, and magnetic hyperthermia [25]. Among spinel ferrites, ZnFe_2_O_4_ NPs have gained attention in various biomedical applications due to their bio-friendly character, lower toxicity than other metal ferrites, chemical stability, easy and reproducible synthesis, low saturation magnetization, and photo-induced catalytic reactant properties [26, 27].

Owing to many advantages in biomedical applications, it is worth exploring the wound healing potential of ZnFe_2_O_4_ NPs and thoroughly investigate their anti-bacterial efficacy and mechanism of action in burn wound bactericidal effect. In this study, we fabricated ZnFe_2_O_4_ NPs using the co-precipitation process. The co-precipitation method is one of the most convenient, eco-friendly, and economical methods of synthesis, yielding NPs with high purity [28]. However, adequate control is needed to monitor the size, stoichiometry, phase purity, and crystallinity of the NPs [29], which could be controlled in this study by tuning the reaction’s parameters. We further characterized the structure, morphology, and size of the synthesized ZnFe_2_O_4_ NPs using different physicochemical techniques, and also investigated their cytocompatibility and hemocompatibility in a dose-dependent manner. Moreover, we studied the dose-dependent antimicrobial activity of ZnFe_2_O_4_ NPs against selected Gram-positive and Gram-negative microbial strains as possible infection models, exploring the antimicrobial mode of action via different techniques. In addition, by performing a scratch assay, we could further demonstrate the *in vitro* wound healing potential of ZnFe_2_O_4_ NPs. We anticipate that this research will offer solid grounds to use ZnFe_2_O_4_ NPs as a potential source to control bacterial infections in a cost-effective manner, as well as improve the wound healing process.

## Materials and methods

### Chemicals and reagents

Zinc chloride (ZnCl_2_), iron chloride hexahydrate (FeCl_3_.6H_2_O), sodium hydroxide (NaOH), oleic acid, and nutrient agar were purchased from Daejung Korea (Nakdong-daero, Sasang-gu, Busan, Korea). Dulbecco’s Modified Eagle’s Medium (DMEM containing L-Glutamine and 4.5 g/L Glucose), fetal bovine serum (FBS), 100X Penicillin-Streptomycin (Pen/Strep), 0.25 % trypsin-EDTA, silver sulfadiazine, tetracycline hydrochloride, dimethyl sulfoxide (DMSO), Drabkin’s reagent, pure human hemoglobin and bovine serum albumin (BSA) were bought from Sigma-Aldrich (Saint Louis, MO, USA). Bacto tryptic soy broth, Difco Agar, Lb Agar, Live/dead viability/cytotoxicity kit, PrestoBlue dye, polyethylene glycol (PEG), live/dead BacLight bacterial viability kit, and fluorescein isothiocyanate fluorescent dye (FITC) were obtained from Fisher Scientific (Waltham, MA, USA). Tetracycline, Bio-Rad Protein reagent, and dichlorofluorescein diacetate (DCFDA) dye were acquired from Bio-Rad Laboratories (Hercules, CA, USA). NIH-3T3 murine fibroblast, human dermal fibroblast (HDF) cells, and bacterial cultures were provided by American Type Culture Collection (ATCC, Manassas, VA, USA).

### Synthesis of zinc ferrite nanoparticles (ZnFe_2_O_4_ NPs)

ZnFe_2_O_4_ NPs were synthesized via a previously described co-precipitation method with minor modifications [30]. An aqueous solution of iron and zinc chloride was prepared in a fixed 1:2 molar ratio mixture of Zn/Fe. Oleic acid (2 drops in every 75 mL of reaction) was added to the solution as a surfactant. Under constant magnetic stirring, sodium hydroxide (NaOH) solution (3 M) was added dropwise (2 mL/min) until the pH changed to alkaline (> 12). The reaction temperature was maintained at 80 °C during the whole process. After achieving the required level of pH, precipitates were collected via centrifugation (6000 rpm, 5 min) and washed thoroughly with distilled water and 70 % ethanol until the pH turned neutral. After washing, particles were dried at 80 °C to obtain a powder, and finally annealed at 500 °C.

### Characterization of ZnFe_2_O_4_ NPs

The X-ray diffraction (XRD, Bruker, D8 Advanced, Madison, Wisconsin, USA) analysis at the scan rate of 1.2°/min and the 2θ range of 20°–80° was carried out. Cu K_α_ (λ = 1.54056 Å) was used as a radiation source and generated at 40 kV and 40 Ma. The crystallite size was measured using the Debye Scherrer formula [31, 32]. Fourier transform infrared spectroscopy (FTIR) was performed to determine the stretching and bending vibrations of various bonds or functional groups in organic or inorganic materials. FTIR spectra in a range of 400–4000 cm^− 1^ were recorded using the FTIR spectrometer (JASCO FTIR-6600). Field emission scanning electron microscopy (FE-SEM, TESCAN, MIRA3, Institute of Space Technology, ISB) equipped with energy-dispersive X-ray spectroscopy (EDX), was conducted to assess the surface morphology and elemental compositions of ZnFe_2_O_4_ NPs. For this purpose, dried powder of ZnFe_2_O_4_ NPs was sprinkled on double-sided carbon-coated tape, followed by gold sputtering, to improve conductivity during imaging. FE-SEM images were captured under an acceleration voltage of 20 kV.

### Cytocompatibility study

A cytocompatibility study was performed according to the previously reported literature to check the toxicity of ZnFe_2_O_4_ NPs [33]. For this purpose, HDF cells were cultured in DMEM, containing 10 % FBS and 1 % Pen/Strep antibiotics, and incubated in a standard environment of 5 % CO_2_ and 37 ºC. To evaluate metabolic activity of the cells, the PrestoBlue assay was carried out according to the manufacturer’s protocol. The HDFs (5000 cells/mL) were seeded in a 48-well plate and allowed to attach for 24 h. Subsequently, experimental concentrations of ZnFe_2_O_4_ NPs (62, 125, 250, 500, and 1000 µg/mL) were prepared in cell culture media (DMEM) and sonicated for 30 min until completely dispersed. In the concentration range we tested (< 1000ug/mL), the nanoparticles were completely dispersed in the medium and did not precipitate over time. The dispersed NPs were applied to the cells and incubated for 5 days. The PrestoBlue assay was conducted on Day 1 and 5 of the treatment, and fluorescence intensity was quantified using a microplate reader (excitation/emission wavelengths of ~ 530/590 nm, BioTek UV/VIS synergy 2, USA). To monitor cell viability of HDF cells, live/dead assay was performed on day 5 of NPs treatment. The cells were incubated for 20 min with staining solution (1 mL), composed of ethidium homodimer-1 (20 µL) and calcein-AM (5 µL) in DPBS (10 mL). Sample pictures were acquired using an inverted fluorescence microscope (Axio Observer 5, Zeiss, Germany) at excitation/emission wavelengths of 528/617 nm and 494/515 nm for ethidium homodimer-1 and calcein, respectively.

### In vitro hemolysis assay

Hemolytic activity of ZnFe_2_O_4_ NPs at different concentrations (62, 125, 250, 500, and 1000 µg/mL in DPBS) was evaluated following the Standard Practice for Assessment of Hemolytic Properties of Materials from American Society for Testing and Materials (ASTM E2524-08 guideline (2013)) [34]. Briefly, heparinized fresh human blood was diluted to adjust the hemoglobin level ~ 10 mg/mL. The concentration of hemoglobin was measured by Drabkin’s reagent, using a standard curve generated from the known concentrations of pure human hemoglobin. Subsequently, the experimental concentrations of NPs were added to 800 µL DPBS in Eppendorf tubes and gently mixed with 100 µL of the diluted blood. Triton X-100 (1 % v/v in DPBS) and PEG (4.4 % v/v in DPBS) were used as positive control (PC) and negative control (NC), respectively. The samples were placed at 37 °C water bath for ~ 3 h and then centrifuged (14,000 rpm, 15 min) at room temperature. The supernatant (100 µL) was added to 100 µL Drabkin’s reagent in a 96-well plate, and was shaken gently for 15 min, in the dark. The absorbance at 540 nm was measured using a microplate reader. Percent hemolysis was assessed using the below equation:

$${\text{Hemolysis} (\% ) = \text{(Hemoglobin\,concentration in sample/Total diluted blood hemoglobin)}} \times {{100}}$$

### Antimicrobial test

The antimicrobial activity of ZnFe_2_O_4_ NPs was performed following a standard agar well diffusion method, with minor modifications [35]. The bacteria selection was made according to the prevalence at burn wound infection site [36, 37]. Selected concentrations of ZnFe_2_O_4_ NPs for antimicrobial activity were 12.5, 25, 50, and 100 µg/mL. Firstly, an inoculum of selected microorganisms was added in autoclaved nutrient broth, and optical density (OD) was maintained between 0.1 and 0.5 at 600 nm, using nanodrop (OneC Microvolume UV-Vis Spectrophotometer, Thermo Scientific, USA) similar to that explained elsewhere [38–41]. This inoculum was evenly spread on agar plates. Wells (8 mm in diameter) were bored on an inoculated agar plate, and the NPs solution (100 µL) was poured into the well and incubated at 37 °C for 12 h. Sterilized 8 mm filter paper disk loaded with silver sulfadiazine cream (15 µg/disk) was placed on plates, which acted as a positive control, and DMSO was poured in one well as vehicle control. The zone of inhibition was evaluated after 24 h.

The antimicrobial activity of ZnFe_2_O_4_ NPs was further confirmed by live/dead bacterial viability kit using one representative Gram-negative bacteria (*E. coli)* and one Gram-positive bacteria (*S. aureus*), respectively. Only one representative concentration (100 µg/mL) of ZnFe_2_O_4_ NPs was tested, as this concentration was the highest dose to exhibit no toxicity when tested with human cells. The live/dead assay was performed following a previously reported protocol [42]. The *E. coli* and *S. aureus* cultures were grown overnight until log phase was reached and a concentration of 1 × 10^5^ CFU/mL was ensured. The selected concentration (100 µg/mL) of ZnFe_2_O_4_ NPs were added to the bacterial strains and incubated at 37 °C for 8 h. Bacterial strains incubated with tetracycline were considered as a positive control. Following incubation, bacteria were collected via centrifugation (10,000 rpm, 5 min, 4 °C). Resultant pallets were washed with DPBS and stained for 15 min under a dark condition with SYTO9 and propidium iodide (PI). A fluorescence microscope captured fluorescence images of the stained bacteria at an excitation wavelength of 540–580 nm and an emission of 600–660 nm for PI. The excitation wavelength of 465–495 nm, and emission of 515–555 nm were used for SYTO9.

### Membrane permeability assay

The membrane permeability assay was conducted following the reported protocol [43]. During the exponential phase, bacteria (*E. coli* and *S. aureus*) were cultured and incubated for 12 h with a concentration of 100 µg/mL ZnFe_2_O_4_ NPs in LB growth medium, containing FITC dye (0.05 % w/v). Tetracycline (5 mg/mL) treated bacteria served as a positive control. Excessive FITC dye was removed from the media by several autoclaved distilled water washings and centrifugations. Finally, photographs of the bacteria were taken with a fluorescence microscope at excitation/emission wavelengths of ~ 491/516 nm.

### Protein leakage assay

To check the effect of ZnFe_2_O_4_ NPs treatment on bacterial cell protein leakage, Bio-Rad protein assay was performed following the reported protocol [44]. For this assay, the fresh tryptic soy broth (TSB; Bacto; 5 mL) cultures of *E. coli* and *S. aureus*, were washed with normal saline through centrifugation (10,000 rpm, 15 min). Resultant pellets were suspended in normal saline and treated with 100 µg/mL of ZnFe_2_O_4_ NPs for 8 h at 37 °C. Afterward, bacterial suspension was centrifuged (12,000 rpm, 15 min), and the acquired supernatant was used for protein content estimation via the Bio-Rad protein assay kit, which relies on the Bradford principle. Protein concentration was measured in ZnFe_2_O_4_ NPs treated samples from the standard curve established by known amounts of bovine serum albumin (BSA) and compared with untreated bacteria (control).

### Reactive oxygen species (ROS) measurement

The level of ROS produced in bacteria after treatment with NPs was measured using the previously reported DCFDA dye method [45]. Initially, *E. coli* and *S. aureus* were incubated with 100 µg/mL ZnFe_2_O_4_ NPs for 8 h and then centrifuged for 5 min at 9000 rpm. Resultant pellets were resuspended in DCFDA dye (30 µg/mL in DPBS) and incubated in dark condition for 30 min at 37 ºC. Subsequently, centrifugation was carried out, and the pellet was resuspended in DPBS. Further, fluorescence intensity was quantified at excitation/emission wavelengths of 485/528 nm using a microplate reader.

### In vitro **scratch assay**

The in vitro scratch assay of ZnFe_2_O_4_ NPs was conducted according to the previously reported protocol [46]. For this purpose, NIH-3T3 fibroblasts (1 × 10^6^ cell/mL) were seeded with complete DMEM media in a 6-well plate and incubated at standard conditions. As soon as the cell growth reached the uniform monolayer, a scratch was made with a sterilized pipette tip, followed by cell washing with DMEM (without FBS) to remove excess detached cells. Subsequently, the sterilized ZnFe_2_O_4_ NPs (100 µg/mL in FBS-free DMEM) were added to the cells. In the control group, cells were remained untreated. After 0, 18, and 36 h of treatment, images were taken with bright field microscopy (Axio Observer 5, Zeiss, Germany). Furthermore, the initial and final width of the scratch were measured with ImageJ software (version 1.52e). The scratch shrinkage percentage was calculated with the following formula:

Scratch shrinkage (%) = (Original width – Final width) × 100/Original width.

### Statistical analysis

All data were taken in triplicate, and the mean ± standard deviation (SD) was measured via GraphPad Prism (version 7.03). One-way ANOVA, followed by Tukey’s multiple comparisons test, was used to analyze the data for all the experiments except PrestoBlue and scratch assay’s results which were analyzed with Two-way ANOVA and t-test, respectively. P-value ≤ 0.05 was considered statistically significant.

## Results and discussion

### Analyses of crystal structure and FTIR spectrum of ZnFe_2_O_4_ NPs

**Fig. 1:**
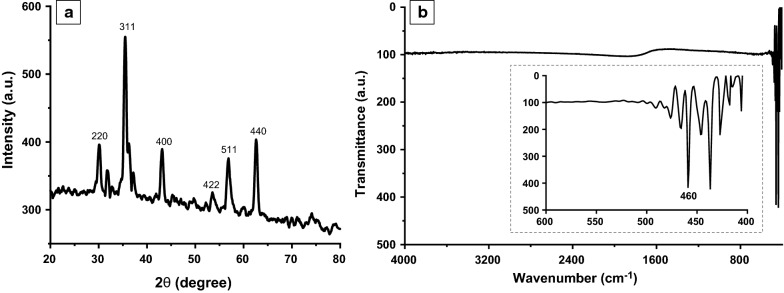
**a** X-ray diffraction (XRD) patterns, **b** Fourier-transform infrared spectroscopy (FTIR) spectrum of the synthesized zinc ferrite (ZnFe_2_O_4_) NPs

First, we fabricated ZnFe_2_O_4_ NPs using co-precipitation process. The co-precipitation method is one of the most convenient, eco-friendly and economical methods of synthesis, yielding NPs with high purity [28]. However, adequate control is needed to monitor the size, stoichiometry, phase purity, and crystallinity of the NPs [29], which could be controlled in this study by tuning the reaction’s parameters. Since the crystalline phase and crystallite size of the NPs are the parameters that influence on their biological activity and properties [47], we analyzed crystal structure and phase formation of ZnFe_2_O_4_ NPs, via XRD spectrum. The XRD analysis is important because it provides information regarding the crystalline structure, nature of the phase, lattice parameters, and crystalline grain size of NPs [48]. As shown in Fig. 1a, the XRD diffraction patterns confirmed synthesis of pure phase and crystalline spinel ferrite NPs. The well-resolved peaks corresponded to the (220), (311), (400), (422), (511), and (440) planes of ZnFe_2_O_4_ (JCPDS: 001-1109), suggesting the successful synthesis of ZnFe_2_O_4_ NPs. These findings are in agreement with previously reported results [49, 50]. The average crystallite size calculated via the Scherer formula was ~ 46 nm. In addition to XRD analysis, we further analyzed the formation of the spinel structure of zinc ferrite and its cation distribution was explored by FTIR analysis [51]. FTIR spectrum of ZnFe_2_O_4_ NPs was recorded in the range of 400–4000 cm^− 1^, as shown in Fig. 1b. The inset of Fig. 1b clearly shows that all the peaks present in the range of 400–600 cm^− 1^, confirming that the capping agent/surfactant used during the synthesis did not bind with ZnFe_2_O_4_ NPs. The absorption band at 460 cm^− 1^ represents the intrinsic metal-oxygen stretching vibration located at the octahedral sublattice (Fe) site. Another absorption band at ~ 500 cm^− 1^ was assigned to metal-oxygen vibration in tetrahedral lattice (M) sites. The observed bands’ positions for ZnFe_2_O_4_ NPs are in agreement with the characteristic vibration bands of spinel nano ferrite [52, 53].

### Surface morphology and chemical compositions of ZnFe_2_O_4_ NPs

**Fig. 2:**
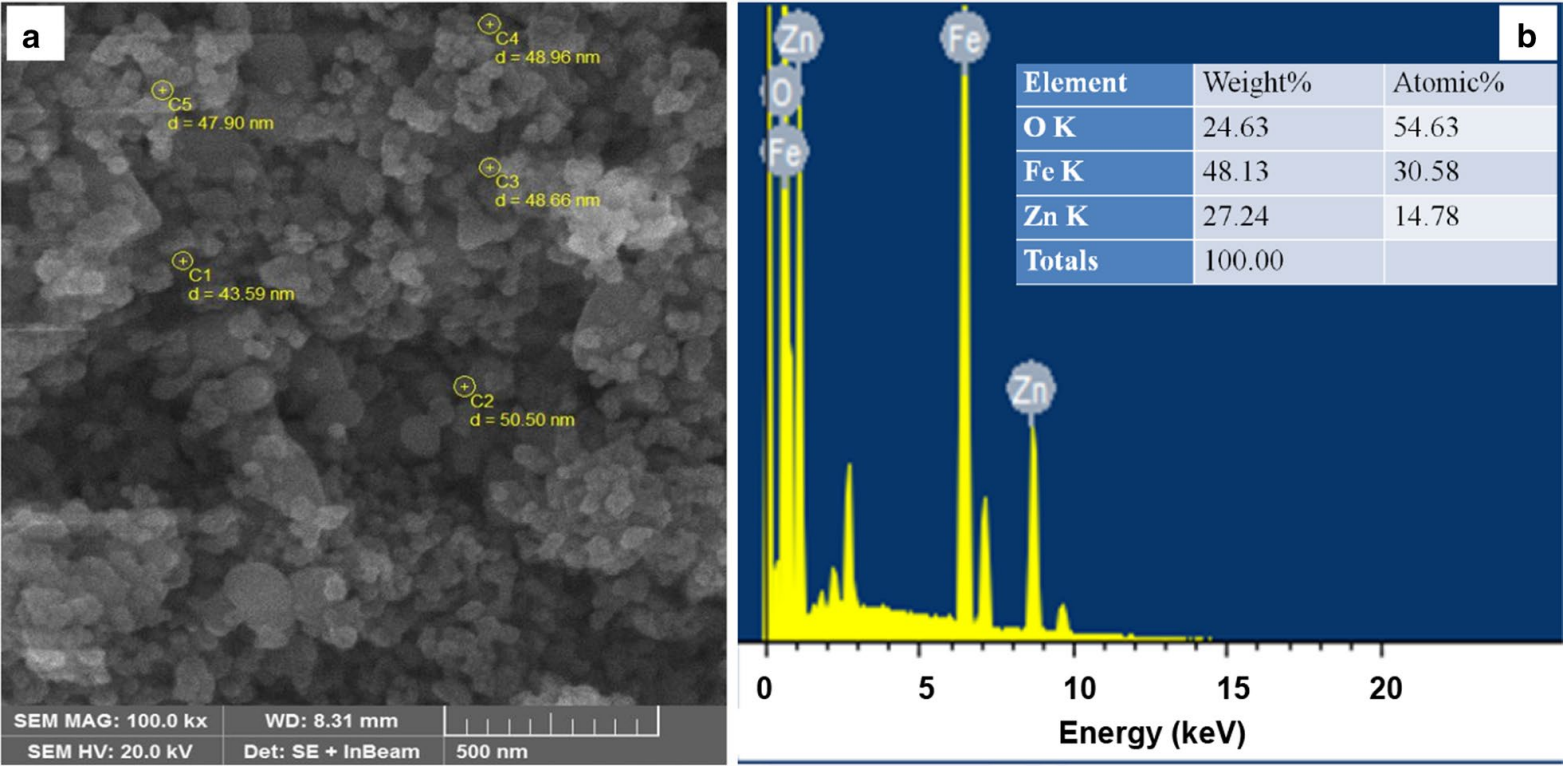
**a** Field-emission scanning electron microscopy (FE-SEM) image of zinc ferrite nanoparticles (ZnFe_2_O_4_ NPs) exhibits spherical particles with an average size of 47.9 ± 2.5 nm. **b** Energy-dispersive X-ray spectroscopy (EDX) spectrum of ZnFe_2_O_4_ NPs shows the elemental compositions reflecting with the determined weight percentage and atomic percentage of all elements (i.e., zinc, Zn; iron, Fe; and oxygen, O) according to stoichiometry

The very small size of spinel ferrites allows them to easily cross the tiny pores of a bacterial cell wall, resulting in a bactericidal effect. The antibacterial activity of spinal ferrites is accredited to the cation attraction by the protein in the bacterial cell wall which leads to the formation of insoluble metal proteinate and associated bacterial death [54]. Among various physico-chemical properties, the size, shape and surface charge of the NPs can affect their internalization and biological activity [55], such as anti-bacterial potential. For example, NPs with a smooth surface have a more chance to contact with a bacterial cell wall [56]. Also, spherical NPs with a smaller size indicates increased anti-bacterial activity compared to the spherical NPs with a larger size due to having a higher surface area [57]. Therefore, we elucidated surface morphology and size of the synthesized ZnFe_2_O_4_ NPs using the FE-SEM technique. Figure 2 presents the FE-SEM micrograph of ZnFe_2_O_4_ NPs with the EDX spectrum. FE-SEM image showed that most of the particles are spherical in shape and smooth on the surface (Fig. 2a). The particles displayed little agglomeration, with an average particle size of 47.9 ± 2.5 nm. The average size estimated by microscopy was in agreement with our XRD data. Figure 2b exhibits the obtained peaks in the EDX spectrum corresponded to oxygen (O), iron (Fe), and zinc (Zn) elements, as expected. The measured weight % were as follows: O (24.63 %), Fe (48.13 %), and Zn (27.24 %). No additional FTIR peaks were observed, suggesting that the synthesized NPs were devoid of impurities. In addition, these results were in line with those of the previously published ZnFe_2_O_4_ NPs [58].

### Dose‐dependent cytotoxicity effect of NPs

**Fig. 3:**
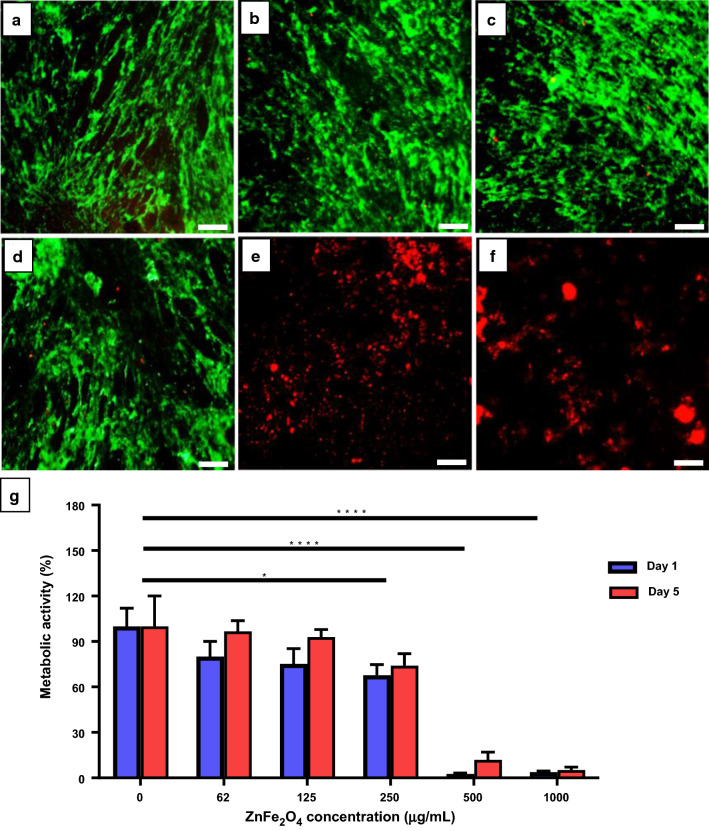
In vitro biocompatibility assay. Representative fluorescence microscopy images of human dermal fibroblast cells (HDF) stained with ethidium homodimer-1 (red color; dead cells) and calcein-AM (green color; live cells) after 5 days incubation with ZnFe_2_O_4_ NPs at concentrations of **a** 0 µg/mL (control), **b** 62 µg/mL, **c** 125 µg/mL, **d** 250 µg/mL, **e** 500 µg/mL, and **f** 1000 µg/mL. Scale bars show 500 µm. **g** The percent (%) metabolic activity of HDF cells on day 1 and 5 after subjecting to different concentrations of ZnFe_2_O_4_ NPs. Error bars denote the standard deviations of 4 replicates, and * represents the significance of the reduction in % metabolic activities of treated cells as compared to control. The data were analyzed by two-way ANOVA, and *p *≤* 0.05, ****p *≤* 0.001 were considered statistically significant

Despite the growing role of NPs in drug delivery and as therapeutic solutions, their medical use is often limited due to toxicity concerns [59]. Therefore, it is important to analyze its biocompatibility with human cells to confirm possible toxicity of ZnFe_2_O_4_ NPs. According to Iacovita et al., ZnFe_2_O_4_ NPs synthesized using polyol mediated procedure (~ 12 nm in size) were biocompatible to human retinal pigment epithelial cells (D407) at the dosage of 100 µg/mL, with reflected viability of 91 %; however by increasing the concentration to 200 µg/mL, cell viability reduced to 50 % [60]. In this study, we evaluated the cytotoxicity of ZnFe_2_O_4_ NP through two methods, a live/dead viability assay that can analyze the live/dead status according to the integrity of the plasma membrane of a cell, and a PrestoBlue assay that can analyze mitochondrial activity. The live/dead viability assay showed that ZnFe_2_O_4_ NPs exhibited dose-dependent toxicity to the HDF cells. At low NPs concentrations (0 µg/mL to 250 µg/mL), no cytotoxic effects were observed in the HDF cells and majority of the cells were green in color (Fig. 3a–d) similar to the control (Fig. 3a), whereas at 500 µg/mL (Fig. 3e) and 1000 µg/mL (Fig. 3f), high numbers of red fluorescence were observed, denoting significant cell death. Through the live/dead assay we were able to observe numerous live cells at doses of ≤ 125 µg/mL, but to confirm that the live cells are not in a state of inhibition of cell proliferation, we further performed the PrestoBlue assay. Similar to the live/dead viability assay, at high NP concentrations of 500 and 1000 µg/mL, the metabolic activity of cells was significantly reduced by 80 % and 95 %, respectively (Fig. 3g). On the other hand, the metabolic activity of 62, 125, and 250 µg/mL of ZnFe_2_O_4_ NPs remained at 96 %, 92 %, and 84 % respectively on day 5 of treatment. Based on these results, we confirmed that a safe dose of ZnFe_2_O_4_ NP for HDF cells was less than 125 µg/mL, as at this concentration more than 90 % of the cells were viable and metabolically active.

The possible mechanisms effecting on cytotoxicity of the MNPs by cells include size, size distribution, shape, surface charge, and route of synthesis [61, 62]. For example, plant-mediated synthesized spherical AgNPs with a size range of 25–40 nm were non-toxic to HCT-116 human colon cancer cells even at the higher dosage of 350 µg/mL, whereas, Algae-mediated synthesized spherical AgNPs with average size of ~ 31 nm cytotoxic against HT-29 colorectal cancer cells [63], demonstrating the effect of synthetic route on toxicity of the NPs. Moreover, the meta study on MNPs revealed that the NPs with the size below 25 nm are more toxic than those with the size range of 25–50 nm against both cancer and normal cells [55].

### Hemocompatibility of NPs with red blood cells

**Fig. 4:**
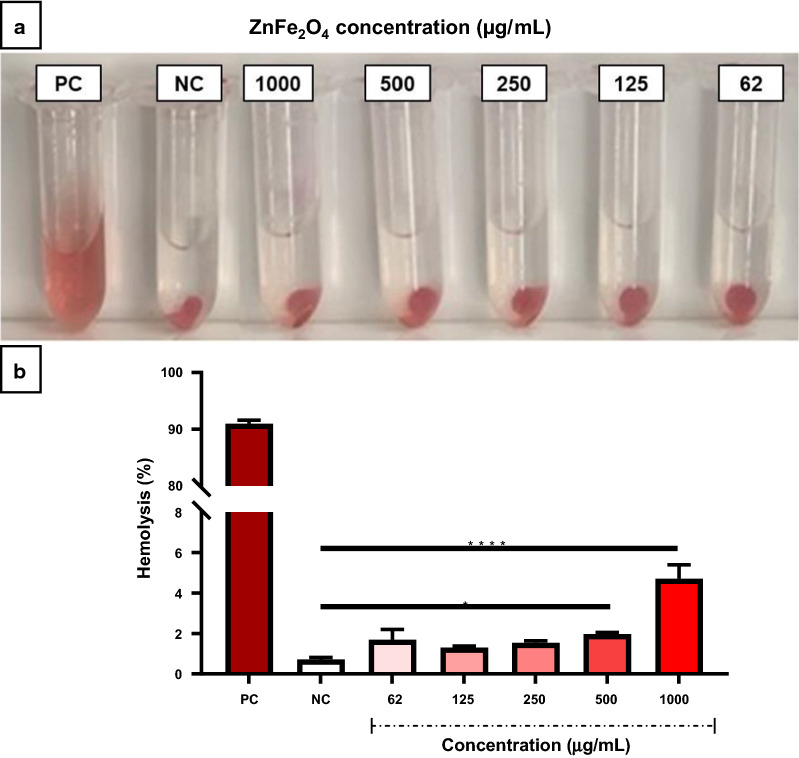
Hemocompatibility of zinc ferrite nanoparticles (ZnFe_2_O_4_ NPs). **a** Photographs of hemolysis assay to detect the presence of hemoglobin in the supernatant of ZnFe_2_O_4_ NPs treated samples. **b** Hemolysis percentage of ZnFe_2_O_4_ NPs treated samples versus positive and negative control. PEG and Triton X-100 lysed blood cells served as a negative control (NC) and positive control (PC), respectively. The values presented in the graph are mean ± SD of triplicate and *p *≤* 0.05, ****p *≤* 0.0001 were considered statistically significant

Along with cytotoxicity testing, we further analyzed the effect of ZnFe_2_O_4_ NPs on human red blood cells. NPs are reported to cause persistent biochemical and morphological changes (erythrocyte membrane rupture) that significantly affect red blood cell function and lead to hemolysis [64, 65]. According to earlier studies on iron oxide NPs, it was found that Fe_2_O_3_ NPs induced 75 % hemolysis at 600 µg/mL and 12.48 % hemolysis at 12.5 µg/mL [66]. Therefore, it is worthwhile to explore the hemocompatibility of ZnFe_2_O_4_ NPs before using them in biomedical applications. The hemolytic activity of ZnFe_2_O_4_ NPs was investigated at different concentrations (62, 125, 250, 500, and 1000 µg/mL). As shown in Fig. 4a, the positive control (i.e., Triton X-100) contained hemoglobin that the color of the supernatant was changed to pink to red color. Interestingly, no hemoglobin was released in the supernatant of the NPs-treated samples and the negative control (i.e., PEG). Quantitative data analysis of free hemoglobin showed no significant hemoglobin release compared to the positive control in NP-treated samples. However, at the concentrations of 500 and 1000 µg/mL, which showed cytotoxicity, slight hemolysis was observed compared to the negative control as shown in Fig. 4b. The amount of hemolysis induced by ZnFe_2_O_4_ NPs was 4.7 % at the highest dose of 1000 µg/mL, which was lower than the allowable tolerance (i.e. 5 %) of the ASTM F756-08 guideline [13]. In addition, our results are in line with the hemolysis results of ZnFe_2_O_4_ NPs reported by Martínez et al. 2019 that ZnFe_2_O_4_ NPs do not cause hemolysis at 200 µg/mL [67]. Our results demonstrate that the addition of a transition metal (zinc) to an iron oxide formulation can significantly improve the hemocompatibility. Based on the cytocompatibility and hemocompatibility results, we used a ZnFe_2_O_4_ NP concentration of less than 125 µg/mL, which did not affect cell viability, in subsequent experiments.

### Bacterial growth inhibition by ZnFe_2_O_4_ NPs

**Fig. 5:**
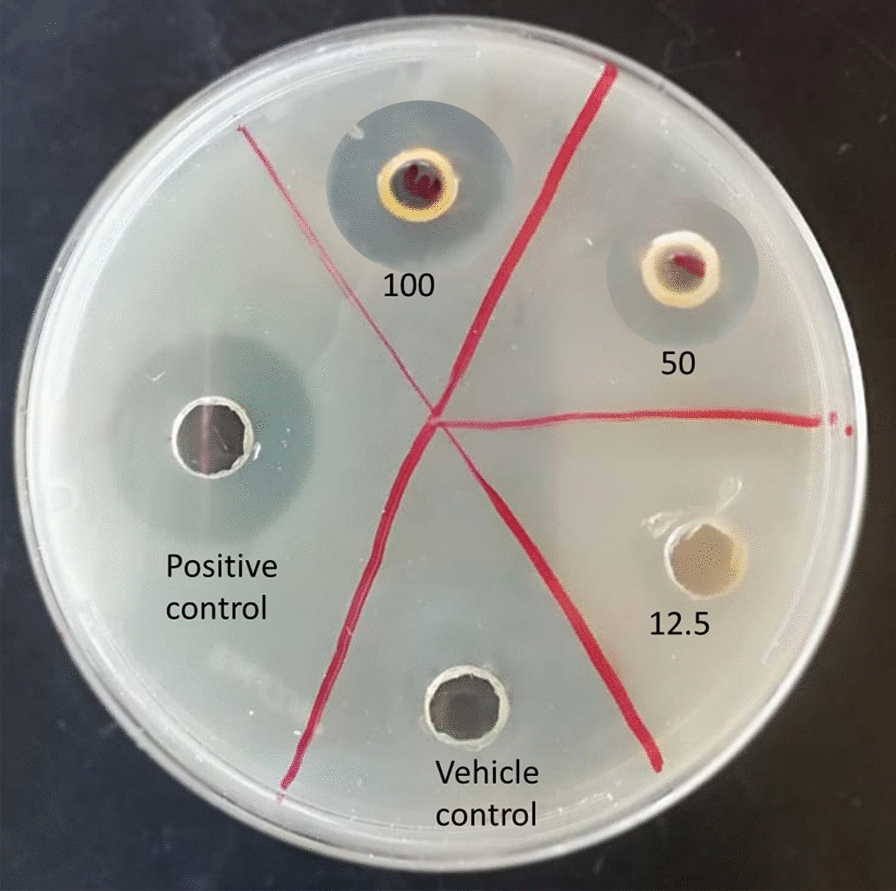
Bacterial growth inhibition of zinc ferrite nanoparticles (ZnFe_2_O_4_ NPs). Representative zone of inhibition images after treatment of *S. aureus* bacteria with different concentrations of ZnFe_2_O_4_ NPs (12.5–100 µg/mL) compared to the vehicle control (DMSO) and positive control (1 % silver sulfadiazine cream)

To verify the antimicrobial effects of ZnFe_2_O_4_ NPs, different concentrations (12.5, 25, 50, and 100 µg/mL) of NPs were applied to four Gram-negative (*E. coli*, *P. aeruginosa*, *S. typhi*, and *K. pneumoniae*) and two Gram-positive (*S. aureus* and MRSA) microbial species which are the most prevalent source of the burn wound infection [36, 37]. In the zone of inhibition analysis test, the higher the sensitivity of the bacterial strain to the anti-bacterial material, the larger the diameter of the bacterial inhibition zone. The representative image of zone of inhibition for *S. aureus* strain is shown in Fig. 5 and the average zone of inhibition for each strain is summarized in Table 1. Our results showed that vehicle control (DMSO) did not inhibit the growth of the bacteria, whereas positive control (1 % silver sulfadiazine cream) exhibited a larger zone of inhibition compared to ZnFe_2_O_4_ NPs treated samples (up to 100 µg/mL of NPs). ZnFe_2_O_4_ NPs showed clear growth inhibitory effects against both Gram-negative and Gram-positive microorganisms in the experimental concentration range (12.5–100 µg/mL). The maximum zone of inhibition was produced against the *S. aureus* (13 ± 0.9 mm) and *K. pneumoniae* (13 ± 1.6 mm) at a concentration of 100 µg/mL NPs. Interestingly, although the maximum amount of ZnFe_2_O_4_ NP used in this experiment was 100 times less than that of the commercially available silver sulfadiazine cream, it showed 68.2–75.9 % bacterial growth inhibitory effects compared to silver sulfadiazine cream. Madhukara et al. demonstrated that ZnFe_2_O_4_ NPs exhibit efficient antimicrobial activity against foodborne pathogens [68]. Similar to our findings, anti-bacterial activity was had previously been reported for ZnFe_2_O_4_ NPs, but only for a single dose (600 µg/mL) [69]. The higher susceptibility of the Gram-positive bacteria to the NPs can be due to the lower stiffness of the cell wall compared to the Gram-negative *E.coli*, which has a complex outer membrane [70]. Another possible reason can be the size, shape, and surface charge of the ZnFe_2_O_4_ NPs, which could render them more favorable to interact with Gram-positive bacteria, which should be studied in the future.

**Table 1:** Estimated zone of inhibition after treatment of pathogenic bacteria with different concentrations of ZnFe_2_O_4_ NPs

ZnFe_2_O_4_ concentration (µg/mL)	Zone of inhibition (mm)					
	*E. coli*	*P. aeruginosa*	*K. pneumoniae*	*S. typhi*	*S. aureus*	MRSA
12.5	9 ± 0.8	10 ± 0.8	10 ± 0.5	8 ± 0.5	10 ± 0.5	10 ± 0.9
25	10 ± 0.4	11 ± 0.4	10 ± 1	10 ± 0.7	10 ± 0.6	10 ± 0.4
50	10 ± 0.6	11 ± 0.5	11 ± 1.1	11 ± 1.4	11 ± 0.8	10 ± 0.7
100	11 ± 0.7	12 ± 0.8	13 ± 1.6	12 ± 1.1	13 ± 0.9	11 ± 0.9
Positive control (silver cream)	16 ± 0.9	15 ± 0.4	16 ± 0.9	16 ± 1	20 ± 1.2	14 ± 0.7
Vehicle control (DMSO)	0 ± 0	0 ± 0	0 ± 0	0 ± 0	0 ± 0	0 ± 0

Data are denoted as mean ± standard deviation of three replicates

**Fig. 6:**
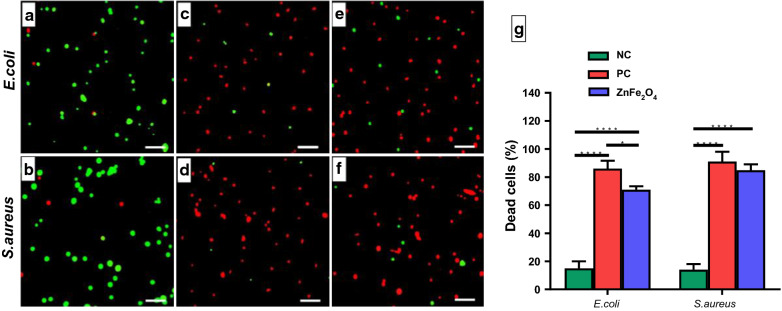
Bacterial live/dead assay. Fluorescence micrographs of *E. coli* and *S. aureus* after treatment with 100 µg/mL of ZnFe_2_O_4_ NPs. **a** Negative control *E. coli* cells without any treatment. **b** *S. aureus* negative control without treatment. **c** *E. coli* treated with tetracycline (positive control). **d** *S. aureus* treated with tetracycline (positive control). **e** *E. coli* treated with ZnFe_2_O_4_ NPs (100 µg/mL). **f** *S. aureus* treated with ZnFe_2_O_4_ NPs (100 µg/mL). **g** Percent (%) dead cells of *E. coli* and *S. aureus* after ZnFe_2_O_4_ NPs treatment as compared to the negative control (NC) and positive control (PC). Experiments were performed in triplicate. The percent dead cells of *E. coli* and *S. aureus* were significantly higher in nanoparticle-treated groups and positive control as compared to the negative control. *p ≤ 0.05 and ****p ≤ 0.0001 are statistically significant. Scale bars show 200 µm

Taking one step further from analyzing bacteriostatic effect of ZnFe_2_O_4_ NPs, the bactericidal effect of NPs was analyzed by live/dead assay. For this assay, one representative Gram-negative bacteria (*E. coli*) and one Gram-positive (*S. aureus*) were selected. SYTO 9, a green fluorescent dye, adheres to healthy living bacterial cells, and PI combines with dead and damaged cells to emit red fluorescence, allowing the evaluation of bacterial death [71]. As shown in Fig. 6, significant bacterial killing was not observed in the *E. coli* and *S. aureus* negative controls (Fig. 6a, b), while most of the bacteria were killed in the positive control treated with 60 µg/mL of tetracycline (Fig. 6c, d). As shown in Fig. 6e and f, *E. coli* and *S. aureus* cells treated with NPs (100 µg/mL) also showed numerous dead cells, emitting red fluorescence. The bactericidal efficacy by NPs was not only significantly higher than the untreated negative control, but was high enough to correspond to the positive control. Quantitative results show that ZnFe_2_O_4_ NP treatment caused 71 % and 85 % of bactericidal effect in *E. coli* and *S. aureus*, respectively (Fig. 6g). The bactericidal efficacy of ZnFe_2_O_4_ NPs was as good as tetracycline and significantly higher than the untreated negative control. The Gram-positive bacteria, *S. aureus* was more susceptible to NPs treatment compared to Gram-negative *E. coli* similar to bacteriostatic results. These findings are in line with previously reported literature indicating that ZnFe_2_O_4_ NPs possess broad-spectrum antimicrobial properties [72, 73]. It has been reported that ZnFe_2_O_4_ NPs can efficiently kill *E. coli* and *S. aureus* at 5 mg/mL doses [73]. However, as can be seen in our cytocompatibility study (toxic at > 200 µg/mL), this reported dose is quite high and may be toxic to mammalian cells as well. In this study, we were able to show that ZnFe_2_O_4_ NPs synthesized by the co-precipitation method showed higher antibacterial activity, which could kill bacteria at a much lower dose (i.e., 100 µg/mL) than other NP synthesis methods.

### Effects of NPs on bacterial cell membrane permeability

**Fig. 7:**
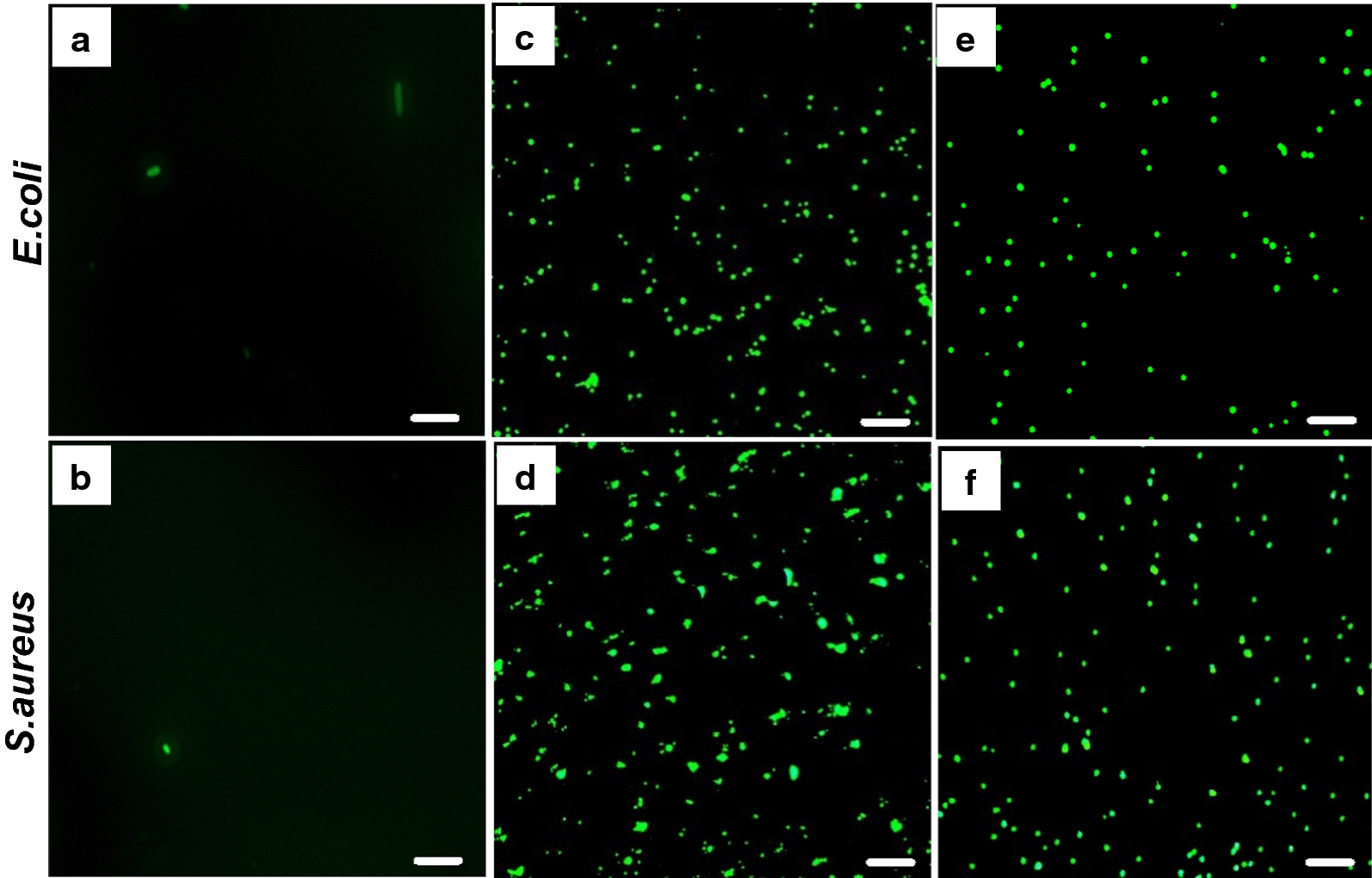
Membrane permeability assay. Fluorescence images of *E. coli* and *S. aureus* showing the influx of FITC after treatment with zinc ferrite (ZnFe_2_O_4_) NPs and tetracycline (positive control). **a** Untreated *E. coli* (negative control). **b** Untreated *S. aureus* (negative control). **c** *E. coli* treated with tetracycline (positive control). **d** *S. aureus* treated with tetracycline (positive control). **e** *E. coli* treated with 100 µg/mL of ZnFe_2_O_4_ NPs. **f** *S. aureus* treated with 100 µg/mL of ZnFe_2_O_4_ NPs. Tetracycline and ZnFe_2_O_4_ NPs induced membrane damage to both Gram-positive and Gram-negative bacteria, resulted in the membrane permeability to the green fluorescent FITC dye. Scale bars show 100 µm

Along with the bacteriostatic and bactericidal effect results, we further explored the effect of NPs on membrane permeability as one of the mechanisms involved in bacterial death. Bacterial cell membrane behaves as a chemical compartment, helping to retain cellular hemostasis, acting as a barrier, and providing selective permeability to the cell, thus playing a significant role in diverse physiological functions. Any disruption in bacterial cell membrane structure adversely affects its function, potentially leading to death [74, 75]. Bacterial cells are generally impermeable to the fluorescent dye FITC, but if their membrane is disrupted by the anti-microbial agent, FITC can quickly penetrate the bacteria and render them fluorescent green. As expected, the negative control did not show a noticeable green fluorescence in *E. coli* and *S. aureus* (Fig. 7a and b), and intense green fluorescent was observed in the positive controls treated with 60 µg/mL tetracycline (Fig. 7c and d). *E. coli* and *S. aureus* treated with ZnFe_2_O_4_ NPs (100 µg/mL) showed prominent fluorescent green, indicating membrane disruption (Fig. 7e and f). Previously, certain NPs, such as oleoyl-chitosan NPs, have been reported to affect membrane permeability through membrane damage in *E. coli* and *S. aureus*. Fei et al., also found that nano-sized silver NP clusters wrinkled and punctured the bacterial membrane, causing significant leakage of cytoplasmic content and eventually causing bacterial death. [76]. Our findings demonstrated that ZnFe_2_O_4_ NPs treatment can cause membrane disruption in both Gram-negative and Gram-positive strains, and can lead to bacterial death through cell membrane damage.

### Effects of ZnFe_2_O_4_ NPs treatment on bacterial protein leakage

**Fig. 8:**
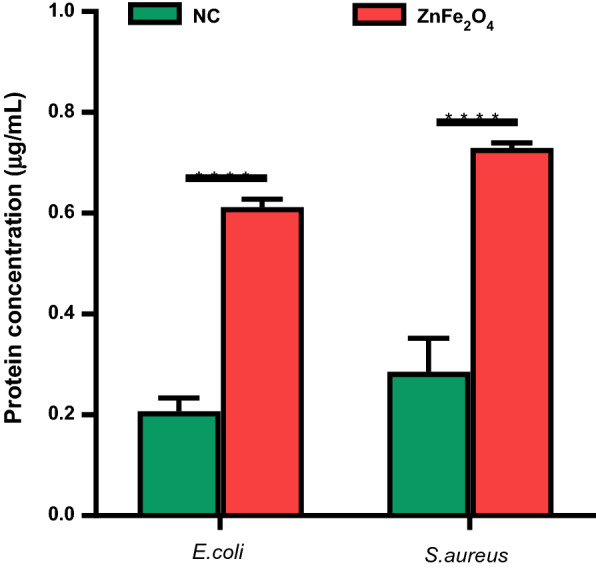
Protein leakage assay. The concentration of protein detected in ZnFe_2_O_4_ NPs (100 µg/mL) treated *E. coli* and *S. aureus* after 8 h of treatment versus untreated bacteria as a negative control (NC). All experiments were done in triplicate, and the data are presented as mean ± SD. ****p ≤ 0.0001 was considered statistically significant

We then investigated whether the disruption of bacterial cell membrane by ZnFe_2_O_4_ NPs could lead to leakage of cytoplasmic materials (e.g., minerals, proteins, and genetic materials). Figure 8 shows the leaked protein concentrations of *E. coli* and *S. aureus* after ZnFe_2_O_4_ NPs treatment compared to positive and negative controls. The NP-treated groups showed protein leakage of 0.6 µg/mL and 0.7 µg/mL, respectively, in *E. coli* and *S. aureus*, which was ~ 3x and ~ 2.5x higher than untreated control. Similar results were observed for zinc oxide (ZnO) NPs, which caused protein leakage in *Acinetobacter baumannii* by damaging the bacterial cell membrane [77]. Silver NPs (AgO) are also reported to trigger membrane damage and protein leakage in both Gram-negative and Gram-positive microorganisms [78]. Despite the higher bacteriostatic and bactericidal effects of ZnFe_2_O_4_ NPs in Gram-positive strains, the protein leakage concentration by Gram-negative bacteria (*E. coli*) was higher than that of Gram-positive bacteria (*S. aureus*), which may be due to differences in the structure and chemical composition of the cell wall. The bacterial wall of Gram-negative bacteria is composed of a specific arrangement of lipid A, lipopolysaccharides and peptidoglycans less than 15 nm thick. However, Gram-positive bacteria contain mainly very thick peptidoglycans with a cell wall of ~ 80 nm, which acts as a boundary layer, protecting the large molecules such as protein to be easily leaked out after disruption of the cell membrane [79].

### ROS generation after treatment with NPs

**Fig. 9:**
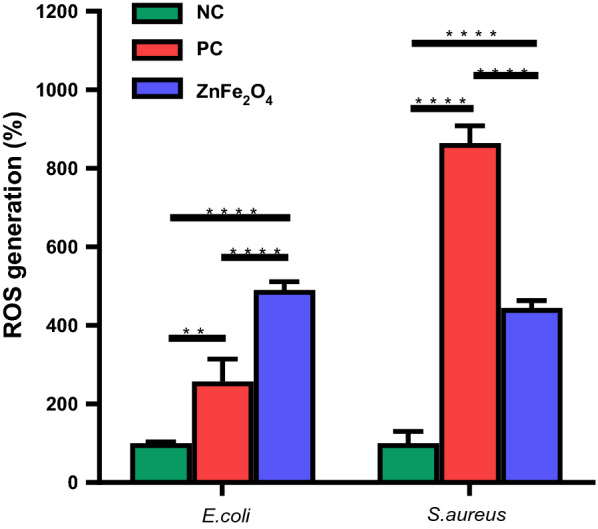
Reactive oxygen species (ROS) assay. Intracellular ROS production after 8 h of treatment with ZnFe_2_O_4_ NPs (100 µg/mL) in *E. coli* and *S. aureus*. Hydrogen peroxide (H_2_O_2_) treated cells were taken as a positive control (PC), and bacteria without treatment served as a negative control (NC). All experiments were performed in triplicates, and data are presented as mean ± SD. **p ≤ 0.01 and****p ≤ 0.0001 were considered as statistically significant

ROS production represents the oxidative stress of bacterial cells, which is one of the mechanisms of bacterial cell death by anti-microbial agents [80]. The adsorption of small nanomaterials on the cell surface can produce intracellular ROS leading to bacterial cell death. Therefore, we analyzed intracellular ROS production in *E. coli* and *S. aureus* treated with ZnFe_2_O_4_ NPs to analyze the bactericidal mechanism of ZnFe_2_O_4_ NPs. As shown in Fig. 9, the ROS production of the NP-treated groups was approximately 5 times higher than that of the untreated group (negative control). Interestingly, ROS production in NP-treated E. coli was ~ 2.5 times higher than that of the positive control, H_2_O_2_ treated *E. coli*. Previously, Dong et al. reported that the ROS generation through treatment with ZnFe_2_O_4_ NPs was sufficient to kill tumor cells [81]. In addition, a hybrid of ZnO/ZnFe_2_O_4_ can also produce ROS in bacterial cells, which is responsible for bacterial cell death [82]. Taken together, our results showed that ZnFe_2_O_4_ NPs can induce ROS production in both Gram-negative and Gram-positive bacteria.

### Enhanced wound shrinkage by ZnFe_2_O_4_ NPs

**Fig. 10:**
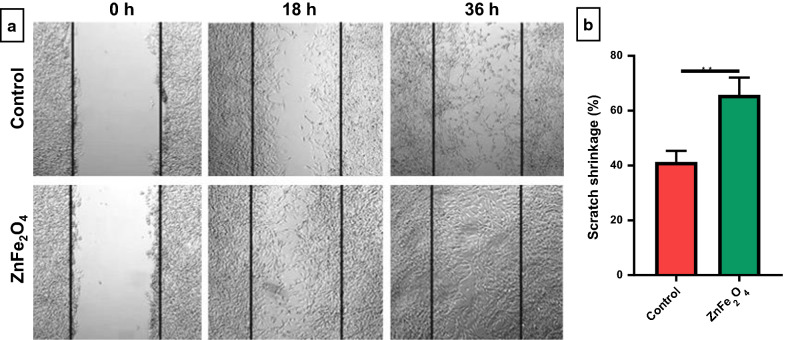
Scratch assay of NIH-3T3 fibroblast cells in the presence of ZnFe_2_O_4_ NPs (100 µg/mL). **a** Bright-field images of the negative control (untreated cells) and ZnFe_2_O_4_ NPs treated cells after 0, 18, and 36 h of incubation. **b** Percent (%) scratch shrinkage of ZnFe_2_O_4_ NPs treated cells versus the untreated control after 18 h. The experiments were performed in triplicates, and data are presented as mean ± SD. The data were analyzed using t-test, where **p ≤ 0.01 was considered statistically significant

Complex wounds such as burns require the application of substances that promote wound healing as well as anti-bacterial properties. The in vitro scratch assay is used to understand the cell migration and proliferation during the wound healing process, and is a useful method for investigating the wound healing potential of drugs or nanoparticles [83]. This assay can conclusively reveal whether a specific compound or substance accelerates or slows down the rate of cell migration and proliferation [84]. In vitro scratch assay was performed to demonstrate the wound healing potential of ZnFe_2_O_4_ NPs at a biocompatible dose (100 µg/mL). As can be seen in Fig. 10a, improved cell migration and wound closure were observed in ZnFe_2_O_4_ NPs treated samples compared to the untreated control after 18 h and 36 h of treatment. In addition, significantly higher percent scratch shrinkage (66 %) was observed in NPs treated samples compared to the control (41 %) at 18 h post-treatment (Fig. 10b). Previously, bacterial nanocellulose/iron oxide (BCN/Fe_3_O_4_) nanofilms treatment showed improved HDF migration in in vitro scratch assay [83]. Mirzahosseinipour et al. also found that curcumin-Silica NPs improve HDF cell migration and proliferation [85]. The silver-incorporated cotton fabrics were also exhibited significant in vivo wound healing effect [86]. Similarly, Boomi et al. observed accelerated wound healing effect in diabetic BALB/c mice after topical application of gold nanoparticles (AuNPs) on the surface of wound [87]. The possible mechanisms of MNPs-induced wound-healing proposed include enhanced angiogenesis, modifying the membrane potential, preventing enzyme ATP synthase, and generating intracellular ROS which results in hampered energy metabolism and wound healing. To the best of our knowledge, this is the first study conducted to evaluate ZnFe_2_O_4_ NPs wound healing potential. Taken together, our research provided the basis for further wound healing applications of ZnFe_2_O_4_ NPs as well as their promising anti-bacterial effect.

## Conclusions

ZnFe_2_O_4_ NPs synthesized by co-precipitation method are spherical in shape, show little agglomeration, have uniform elemental compositions, and possess all characteristic peaks of spinel ferrite NPs. The prepared ZnFe_2_O_4_ NPs presented excellent biocompatibility and hemocompatibility with HDFs and human RBCs and exhibited potent antimicrobial activity against both Gram-positive and Gram-negative microbial strains at biocompatible concentration. We also revealed the possible antimicrobial mechanisms that ZnFe_2_O_4_ NPs triggered bacterial cell death via membranes disruption, protein leakage, and ROS generation for the bactericidal efficacy. Furthermore, our NPs also exhibited excellent *in vitro* wound healing properties. This study showed that ZnFe_2_O_4_ NPs have promising antibacterial properties and desirable biocompatibility to promote wound healing. Hence, the optimized ZnFe_2_O_4_ NPs can be used as an alternative therapeutic agent against drug-resistance microbial pathogens and also as a bandage to enhance burn-wound healing in near future. In addition, future study is required to investigate short-term and long-term toxicity and wound-healing effect of ZnFe_2_O_4_ NPs in animal models.

## Data Availability

All the generated or analyzed data during this study are included in this manuscript.
